# Patients’ symptoms and challenges during the first year after allogeneic haematopoietic cell transplantation—patients’ own expressions

**DOI:** 10.1007/s00520-026-10655-5

**Published:** 2026-04-15

**Authors:** Carina Lundh Hagelin, Annika M. Kisch, Katarina Holmberg, Anna O’Sullivan, Yvonne Wengström, Jeanette Winterling, Karin Bergkvist

**Affiliations:** 1 Department of Health Care Sciences, Marie Cederschiöld University, P.O 11189, SE-100 61 Stockholm, Sweden; 2 Department of Neurobiology, Care Sciences and Society, Division of Nursing, Karolinska Institutet, Stockholm, Sweden; 3 Department of Hematology, Oncology and Radiation Physics, Skane University Hospital, SE-221 85 Lund, Sweden; 4 Department of Health Sciences, Lund University, Lund, Sweden; 5 Department of Nursing Care, Sophiahemmet University, SE 114 86 Stockholm, Sweden; 6 Department of Nursing, Sophiahemmet University, Stockholm, Sweden; 7 ME Breast, Endocrine and Sarcoma Cancer, Karolinska Comprehensive Cancer Centre, Stockholm, Sweden; 8 ME Head & Neck, Lung & Skin Cancer, Karolinska Comprehensive Cancer Centre, Stockholm, Sweden; 9 Department of HHLH, Karolinska Institutet, Stockholm, Sweden; 10 Department of Public Health and Caring Sciences, Uppsala University, Uppsala, Sweden

**Keywords:** Allogeneic haematopoietic cell transplantation, Cancer, Cancer survivorship, Symptom occurrence, Symptom experiences, Multidimensional challenge

## Abstract

**Purpose:**

The study aimed to describe patients’ experiences of physical, psychological, social, and existential symptoms and challenges during the first year after allogeneic haematopoietic cell transplantation (allo-HCT).

**Methods:**

Quantitative and qualitative data were collected and analysed with descriptive methods as part of a large retrospective cross-sectional online survey of patients who underwent allo-HCT between 2017 and 2020.

**Results:**

In total, 126 (59.7%) of all eligible patients participated, mean age 57 years (range 21–75), equal distribution of gender and time after allo-HCT. Most patients (58%) had experienced fewer than 5 coexisting symptoms, although 8% reported more than 10. Physical symptoms or challenges (83.0%) were most often reported, followed by social (52%), existential (50%), and psychological (43%), as described in responses to open-ended questions. Analysed descriptions resulted in 12 categories/areas of physical symptoms and challenges; two categories each of psychological—*My inner self* and *Me in my context*; social—*Social isolation* and *Impact on relationships*; and existential symptoms and challenges*—Re-evaluating life and myself* and *An uncertain future*.

**Conclusion:**

Patients experienced a variety of symptoms and challenges as a multidimensional experience during the first year after allo-HCT. A holistic approach to symptoms and challenges is needed to address individual patients’ situations. This knowledge emphasises the importance of patients receiving supportive care to navigate life after transplantation and into survivorship.

## Introduction

Allogeneic haematopoietic cell transplantation (allo-HCT) is an established treatment modality used mainly for haematological malignancies [1, 2]. Over the past decade, allo-HCT has increased, with more than 80,000 procedures performed worldwide each year [3]. Approximately 300 transplantations are performed yearly at six transplant centres in Sweden [4, 5]. Although the goal of treatment is cure, the outcome is rarely predictable [6–8] and patients face considerable risks of morbidity and mortality [9, 10]. Both short- and long-term symptoms occur after allo-HCT and have been shown to affect patients’ quality of life (QoL) and recovery [11–15]. Frequently reported symptoms include fatigue, sleep disturbances, pain, and cognitive limitations [13, 16, 17]. Patients undergoing allo-HCT often experience a prolonged and socially isolating hospitalisation, accompanied by substantial physical and psychological symptom burden [18, 19]. Symptom burden is an overall concept that can be understood as the sum of the occurrence, intensity, and distress of symptoms reported by patients [13, 17]. However, a previously published study [13] found that symptoms such as tiredness, susceptibility to infections, disinterest in sex, and physical weakness were the top four most frequently reported symptoms during the first year after allo-HCT, although the perceived distress associated with these symptoms varied. Following the prolonged period of isolation during treatment, symptoms such as depression and post-traumatic stress disorder (PTSD) have been reported up to six months after transplantation [18, 19]. A life-threatening complication is graft versus host disease (GvHD), which is an immunological reaction between the donated cells and the recipient [20]. GvHD can occur directly after the transplantation (acute GvHD) and up to several years later (chronic GvHD). It can affect all organs, but the main target organs are the skin, gastrointestinal tract, and liver [1]. The effects of GvHD can have a significant impact on visible and persistent physical changes (e.g. skin changes, weight fluctuations and other treatment-related complications) [21, 22] and affect overall body image and impacts patients’ QoL and activities in daily life [23]. Investigating the occurrence of symptoms provides important knowledge about the patient’s situation, while information about symptom-related distress and symptom burden adds to a more holistic perspective [13].

Symptom burden is commonly assessed using structured patient-reported outcome measures (PROMs), with valuable quantitative information about symptom occurrence and severity. However, these instruments may not fully capture how patients themselves perceive, interpret, and describe their symptoms and the challenges they experience in everyday life. This is especially important in the so-called re-entry phase, defined as the first 18 months after a cancer diagnosis and completion of treatment during which patients must pick up life again [24]. A deeper understanding of patients’ own expressions of symptoms and challenges during this phase is therefore needed to inform supportive and person-centred care after an allo-HCT. The exact duration of the 18-month re-entry phase can be difficult to define; focusing on the first year may provide a clearer and more practical timeframe to study.

The aim of the present study was to describe patients’ own experiences and expressions of physical, psychological, social, and existential symptoms and challenges during the first year after allo-HCT.

## Methods

### Study design

The present study employed a retrospective cross-sectional online survey collecting both quantitative and qualitative patient-reported data.

### Setting and study participants

This study focused on patients who had undergone an allo-HCT at the largest allo-HCT centre in Sweden, which performs approximately 100 allo-HCTs per year for both children and adults (4). Inclusion criteria were adult patients (≥ 18 years) who had undergone an allo-HCT between 2017 and 2020, and were able to read and understand Swedish. Exclusion criteria were patients with cognitive impairment or who were too ill to participate.

### Recruitment and data collection

Recruitment was carried out with assistance of the head of nursing development at the centre, who assisted in identifying patients who met the inclusion criteria and to not send invitation letters to people who have died or who fell within the exclusion criteria. The latter by controlling the patients’ medical record. Data were collected from October to December 2021. An invitation to participate was sent by mail to 211 patients identified as eligible. The invitation included written information about the study, contact information to the researchers for more information or if any intrusive thoughts arose, and information about confidentiality. The information also included the right to withdraw from the study at any time without providing a reason, and that doing so would in no way affect their current or future care from their healthcare provider. A QR code and a link to the online survey were included. A paper version of the survey could be sent to patients on request. Patients completed the survey anonymously and only the head of nursing development at the unit had access to the participants’ identities for mailings and reminders. Two reminders were sent. Consent was obtained from participants at the time of completing the survey, either online or via a paper version. The study was approved by the head of the allo-HCT centre and by the Swedish Ethical Review Authority, Dnr: 2020–03996; 2021; 2021–03865.

### Sources of data

Data for the present study included questions about demographics, medical data, and of occurrence and frequencies of symptoms, generating quantitative data. Four separate open-ended questions were used to collect patients’ own descriptions of physical, psychological, social, and existential symptoms and challenges, generating qualitative data. The questions were posed as follows: “Describe in your own words the physical/psychological/social/existential symptoms or challenges you have experienced during the first year after allo-HCT.” The survey also included PROMs for QoL, transition in the care system, person-centred care, and self-care, which will be or has been reported elsewhere [25]. There was no word limit for responses to the open-ended questions. The basis for the survey, including the open-ended questions, was tested with two patient representatives from a patient from the Swedish blood cancer society for relevance and clarity. This was positively supported and did not result in any changes.

### Analysis

Quantitative data about occurrence and frequency of symptoms were analysed using descriptive statistics for mean (m), standard deviation (SD), and percentage (%). Data from the open-ended questions are presented as descriptive text. Combining data sources allowed for a more comprehensive understanding of patients’ personal experiences and perceptions. Missing data are reported as the number of non-responses per question, as the items are not part of a structured scale, making further statistical handling inappropriate. The open-ended questions were analysed using qualitative content analysis, as described by Graneheim and Lundman [26]. First, we analysed the manifest content of each question, i.e. the described symptom or challenges in the physical, psychological, social, or existential dimensions. Analysing patients’ descriptions of physical symptoms and challenges, it became clear that these were mostly connected to different organ systems and were therefore coded and categorised following European Society for Blood and Marrow Transplantation (EBMT) Handbook of Hematopoietic Cell Transplantation and Cellular Therapies [27]. Thereafter we used an inductive and latent approach for analysing data within each dimension. Two authors (CLH and KB) first read all answers, ranging from a few words to more narrative descriptions of up to 237 words. The text was condensed into meaning units comprising words, sentences, or paragraphs relevant to the aim of the study and preserving their core meaning. The condensed meaning units were coded by their inductive content, and, in a next step, codes were grouped together and labelled by their categorical content. In all stages, the two authors discussed the content and sorting of codes and categories to obtain a coherent understanding. When the categories were finally formulated, a third author (AK) confirmed the formulated categories and went back to the raw data to validate the analysis. Data were also discussed within the entire research team to gain an understanding of the results, and for credibility and trustworthiness. The analysis of qualitative data was conducted on the patients’ own statements, which were written in Swedish, and all steps of the content analysis were carried out in Swedish. Translation into English took place once the categories had been created. All categories and quotations have undergone language editing. SPSS statistics version 28.0.1.1 was used for the analysis of quantitative data and Microsoft 365 Excel was used for sorting qualitative data for analysis.

## Results

In total, 126 (59.7%) of all eligible patients participated in the study with an equal distribution of men and women (52 vs 48% respectively) and number of years since allo-HCT (Table 1). Participants were between 21 and 75 years (m = 57, SD = 12.9) and most were married or living with a partner (79%). About 62% were working at the time of the study and 33% reported being on sick leave, either full-time or part-time. The vast majority reported experiences of GvHD (67.5%) and 52% reported having had emergency contacts with the allo-HCT centre during the first year after allo-HCT; 33% reported having been re-admitted for in-patient care (Table 1).

**Table 1:** Patient characteristics (*n* = 126)

		Missing
**Age,** mean (standard deviation)	57 (12.9)	4
	**n (%)**	
**Sex**		1
Male	65 (51.6)	
Female	60 (47.6)	
**Marital status**		1
Married/having a partner	99 (79.2)	
Single	26 (20.8)	
**Children living at home**		
Yes	37 (29.4)	
No	89 (70.6)	
**Education**		
Elementary/secondary school	67 (53.2)	
Higher education	59 (46.8)	
**Working**		
Yes	78 (61.9)	
No	48 (38.1)	
**On sick leave**		
Yes	42 (33.3)	
No	84 (66.7)	
**GvHD**		
Yes	85 (67.5)	
No	41 (32.5)	
**Years since transplantation**		6
2017	28 (23.3)	
2018	29 (24.2)	
2019	29 (24.2)	
2020	34 (28.3)	
**Re-admission since allo-HCT**		1
Yes	41 (32.8)	
No	84 (67.2)	
**Emergency contact with the allo-HCT centre**		
Yes	72 (57.1)	
No	54 (42.9)	

*allo-HCT*, allogeneic haematopoietic cell transplantation. Missing represents the number of patients not answering the specific part

### Occurrence and frequencies of symptoms and challenges

Most patients (*n* = 72, 57%) reported experiencing fewer than 5 simultaneous symptoms during the first year after transplantation, although 32% reported 5–10, and 8% more than 10 concurrent symptoms during the same period (Table 2). These were mostly physical symptoms or challenges (81.0%), followed by social (54.7%), psychological (42.1%), and existential (46.9%) symptoms (Table 2). Between 4.8 and 12.7% of the participants had experienced these symptoms or challenges very often (Table 2).

**Table 2:** Reported symptom occurrence and reported frequencies of symptoms and challenges during the first year after allo-HCT (*n* = 126)

Reported symptom occurrence	< 5 *n* (%)	5–10 *n* (%)	> 10 *n* (%)	Missing *n* (%)
Number of simultaneous symptoms	72 (57.1)	41 (32.5)	10 (7.9)	3 (2.4)
Frequencies of symptoms or challenges reported in the four dimensions	No *n* (%)	Yes, some *n* (%)	Yes many/very often *n* (%)	Missing *n* (%)
Physical	22 (17.7)	86 (68.3)	16 (12.7)	2 (1.6)
Psychological	73 (57.9)	46 (36.5)	7 (5.6)	-
Social	57 (45.2)	57 (45.2)	12 (9.5)	-
Existential	62 (49.2)	53 (42.1)	6 (4.8)	5 (4)

*allo-HCT*, allogeneic haematopoietic cell transplantation. Missing represents the number of patients not answering the specific part

### Patients’ expressions of symptoms or challenges during the first year after allo-HCT

In the open-ended questions, between 54 and 105 patients described experiences of symptoms or challenges in at least one of the four dimensions. These were mostly physical (*n* = 105, 83.33%), followed by social (*n* = 66, 52.38%), existential (*n* = 63, 50%), and psychological (*n* = 54, 42.86%). The descriptions ranged from single words to descriptions of a multidimensional experience. Patients described the same symptom or challenge in more than one of the dimensions, e.g. fatigue as both a physical, psychological, and social challenge.

### Patients’ expressions of physical symptoms and challenges

When analysing patients’ descriptions of physical symptoms and challenges, it became clear that these were mostly connected to different organ systems and were therefore coded and categorised following the EBMT Handbook of Specific organ complications (Table 3). However, there were descriptions that could not be classified according to the handbook, and these were categorised based on their overall content. These were fatigue, GvHD, infections, pain, sleep, and some other descriptions. The descriptions are the patient’s own words, and it should be noticed that e.g. fatigue was expressed as a specific term, but also in ways that are included in common fatigue definitions and therefore classified together. Further, GvHD was expressed as a single word with no physical location mentioned; it was categorised solely as GvHD; however, when a specific location was mentioned, such as the skin or the GI tract, it was categorised in that area (Table 3). Experiences of symptoms or challenges regarding sleep, which might not be related to a specific organ but were often expressed, were given a separate category (Table 3). Pain was described in clear terms, both in general and in relation to a specific location.

**Table 3:** Areas of physical symptoms or challenges one year after allo-HCT as expressed in own words by patients, in alphabetic order following the EBMT Handbook [27]

Specific organ	Patients own words
**Cardiovascular**	blood clots in the legs and lungs, heart challenges such as heart failure and fibrillation, changes in blood pressure - both hyper- and hypotension, oedema
**Endocrine, fertility & sexual health**	menopausal symptoms, hypo- och hyperthyroidism, diabetes, blood in semen, gynaecological challenges, genital ulcers
**Gastrointestinal**	gastric catarrh, stomach ache, nausea, vomiting, diarrhoea, constipation, loss of appetite, and weight loss
**Musculoskeletal**	muscle weakness, difficulties getting muscles working, skeletal and joint pain, vertebral fracture and compression, osteonecrosis, osteoporosis, tendon ruptures, septic arthritis, osteoarthritis, limited mobility, body stiffness
**Neurological**	poor memory, neuropathy, warm and numb feet, dizziness, challenges with balance, difficulty concentrating
**Noninfectious pulmonary**	shortness of breath
**Ocular**	tunnel vision, blurred sight, dry eyes, eye irritation, inflammation of the eyelids, vision challenges, sensitivity to sunlight
**Oral**	dry mouth, blisters, ulcers, and fungus in the mouth, no sense of taste or smell
**Skin**	skin challenges, dry skin, itching, skin reaction, rash, skin challenges on the feet, nail loss, necrosis, hyperpigmentation
*Fatigue*	fatigue, being tired, weak, listless or exhausted, feeling weak, reduced strength, poor fitness, brain fatigue
*GvHD*	GvHD expressed as a single term
*Infections*	pneumonia, sepsis, herpes, shingles, fever, susceptible to infection, UVI, influenza, encephalitis, leg ulcers, fungus
*Pain*	pain, pain under the ribs and in the chest, pain in arms, hands, legs and feet, extreme pain and cramps in the arms and legs, headache, pain in hands and feet, pain in the body, back pain, joint pain
*Sleep*	sleeping challenges, difficulty sleeping, difficulty sleeping due to cortisone treatment
*Other expressions*	dry and fragile mucous membranes, adhesions in the abdomen, nosebleed, loss of hearing

*GvHD*, graft versus host disease. The classification in rows in bold follows the EBMT Handbook of Specific organ complications, in rows in italics are descriptions by patients not able to classify

### Patients’ expressions of psychological symptoms and challenges

The analysis revealed two categories of psychological symptoms or challenges: *My inner self* and *Me in my context.*

*My inner self* included experiences of emotions, fluctuating from feeling happy and grateful to angry, scared, or sad. Feelings of being confused, having mood swings, and being stressed, as well as experiences of anxiety, being in a low mood, or depressed were also reported. Some patients described experiences of intrusive thoughts, death anxiety, and suicidal thoughts. One patient wrote:

A lot of anxiety especially in the evenings and nights. Feeling of meaninglessness, strong concerns about the future and relapse, suicidal thoughts. (pt. no. 30)

Panic attacks were described related to being isolated:

I had anxiety and panic attacks because I felt trapped. A few months after the transplant, I stopped using social media because I felt so bad when I saw other people’s “normal” lives. I also started having extreme anxiety and sometimes panic attacks when talking to strangers. (pt. no 8)

Patients described fatigue as a psychological symptom or challenge, and as something affecting their inner self through its cognitive impact, with difficulty concentrating and remembering things. Sleep challenges were also reported to affect one’s inner self with difficulties sleeping and an altered circadian rhythm.

The category *Me in my context* included experiences of relationship to others. Experiences of being abandoned by others and therefore feeling lonely were described. However, patients also wanted to be alone, often related to the risk of being infected. This was especially connected to the Covid pandemic. Patients described experiencing feelings of guilt about being a survivor when others, patients or friends, had not survived, and of being a burden to the family from being sick and during rehabilitation.

### Patients’ expressions of social symptoms and challenges

The analysis revealed two categories of social symptoms or challenges: *Social isolation* and *Impact on relationships*.

*Social isolation* described patients’ experiences of being isolated due to their compromised immune system. Patients also described wanting to be alone because they did not want to get close to people due to the risk of being infected. Some patients described the social isolation as being primarily in the initial phase and that after a few months, they had the strength to interact with some people again but kept the number of personal contacts low. Other patients chose to stay socially isolated for a long period due to a fear of being infected. The social isolation resulted in an inability to work, experiences of loneliness, boredom, not being able to move around in society as usual, restrictions on going to restaurants, or meeting their children and grandchildren. Being isolated affected the patients’ confidence and their sense of security, described as follows:

Difficult to participate in social settings with several people involved…, with immunosuppression - almost completely isolated. Met very few people the first few years then came Covid. Has affected self-confidence and inner security. (pt. no 14)

Fatigue was also a factor resulting in social isolation due to being too tired to meet friends, or having difficulty concentrating, memory loss, or sensitivity to sound. Patients were affected in their ability to work, including difficulties coping with and managing tasks they had previously performed. Several patients described how not being able to work affected their financial situation. As a result, living conditions and social contacts were affected.

*Impact on relationships* was described in relation to children, partner, family, friends, and healthcare personnel. The impact on relationships with children was associated with not being there for the children, not being able to be part of their lives, having to hand over the responsibility for children to someone else during the treatment trajectory, or when a child had to take responsibility for the household. This was particularly challenging for single parents. The impact on relationships with children was also described as fearful and included a need for psychological support, both during the transplant process and afterwards.

Roles and relationships within families changed when patients needed support and a partner had to take on greater responsibility for the family and children in addition to supporting the ill person. This was something that patients expressed both being grateful for but also difficult because the burden was placed on the other party. Feelings of being a burden to oneself and the family were described:

Several times I’ve felt that it was best for everyone if I did not survive, both for my family and myself, not having to live with the constant worry and anxiety about relapse (pt. no 42).

The experience of being a young adult and dependent on support from a parent was described, both in terms of practical and financial dependence, and feelings of guilt about needing help. Changes in the relationship with a partner and how they cope as a couple in the situation were described. One patient expressed this as a “completely new situation in the relationship with my wife” (pt. no 4). Expressions of challenges regarding intimacy were raised due to an impact on sexuality. The effect on relationships was also connected to difficulties concentrating, and experiences of family and friends not understanding the susceptibility to infection or when they commented that the sick person looked healthy. Participants also described experiencing that the family needed practical support from outside.

### Patients’ expressions of existential symptoms and challenges

The analysis gave two categories of existential symptoms or challenges: *Re-evaluating life and myself* and *My uncertain future.*

*Re-evaluating life and myself* included how patients’ perspectives on life had changed due to being sick and having undergone this curative treatment which gave them an opportunity to choose a new life path, including re-evaluating relationships, described by one patient as:

Thoughts on the meaning of illness. Gratitude for life, and the possibility of changing life’s path. Reconciliation with my ex-husband. (pt. no 19)

Some patients described experiences of desperation, being afraid, and a fear of dying, while others had not felt afraid and had put all their effort into recovery and survival. For help with handling the situation, some patients had sought contact with a counsellor, information material, conversations, and social contacts concerning existential issues. Others, who lacked networks and an ability to seek support by themselves, had desired support from others, e.g. in the form of group support, to better manage their own thoughts and recovery. Some patients did not recognise themselves after the treatment and missed their “old” life and wanted it to become normal again, described as:

Who am I now, I am not cancer free but just don’t have cancer right now. Missed my “old” life very much and wanted to be like I was then, appearance-wise and physically (pt. no 18)

Doubts about whether it had been worth having the treatment were expressed and that they sometimes had a desire to die. There were also expressions of having had thoughts of suicide during the transplantation trajectory. Patients also described experiences of feeling content amidst it all, and that existential thoughts generated good thoughts and the fear of illness and death disappeared.

*My uncertain future* included uncertainties about what would happen in the future, about life and death, and thoughts of the family. Uncertainties also included health and how to live, and about possibilities to work. Patients expressed greater awareness and presence in daily life, doing things in the moment and not waiting. Thoughts about life and death were frequently expressed by patients, often related to their children and of not being around anymore, such as planning for the funeral and leaving something behind for others:

Fear of death and how it would affect my daughter. I wanted to write a lot about how much I loved her so she would know if I wasn’t around (pt. no 26).

## Discussion

By combining quantitative and qualitative data, and by asking patients to describe, in their own words, their physical, psychological, social, and existential experiences after allo-HCT, we have been able to demonstrate the wide range of symptoms and challenges experienced during the first year after allo-HCT. Patients described their experiences on an individual level and also how these had affected their relationships with others, which had transformed them into a new context, demonstrating the challenging situation of survivorship after a cancer diagnosis and treatment [28, 29]. What became clear is the multidimensional nature of symptoms and challenges, where patients described the same experience in several or all dimensions addressed in the survey. Examples of this are fatigue and anxiety, which are described in the physical (fatigue), psychological (both fatigue and anxiety), social (both fatigue and anxiety), and anxiety (existential) dimensions. GVHD can cause many of the symptoms and challenges described, even if it is not specifically referred to by that term. As e.g. GvHD affects the patient’s body image [21, 22] it could further affect social relationships, intimacy, and sexuality [21]. This further highlights the need for a holistic approach when interpreting patients’ expressions of symptoms and challenges after allo-HCT.

Patients described symptoms and challenges that confirm existing knowledge of physical experiences, which could be understood as known medical complications when undergoing allo-HCT [11–15, 30]. Existing knowledge regarding psychological [13], social [30–32], and existential [33–35] symptoms or challenges was also confirmed. However, what the present study adds is patients’ subjective descriptions of their experiences and that the symptoms and challenges are not separate. This means that the patient’s experiences are subjective and form a multidimensional whole [36, 37], demonstrating the importance of understanding the patient’s own perception and interpretation of symptoms and challenges in order to provide the best support. Evidence-based guidelines support rehabilitation assessments and interventions for people with cancer [38] and should incorporate symptoms and challenges expressed by patients themselves. Oncology services should therefore integrate such guidelines into routine care to promote timely referral to rehabilitation and optimise patients’ functioning and QoL [38]. 

Psychological reactions after allo-HCT are well-known [39, 40]. Patients in the present study described these experiences as affecting not only themselves but also the way they think about others in the situation. It has been suggested that psychological reactions after allo-HCT could be classified as PTSD or depression 6 months after allo-HCT [18]. Some patients in the present study described experiencing suicidal thoughts, which has been described earlier [41, 42], and needs to be taken into consideration when caring for these patients. Which professional is best placed to do this may depend on the organisation of care, but this indicates a need for continually monitoring and responding to patients’ experiences, not as separate parts but as a multidimensional whole. This also shows the importance of long-term follow-up after allo-HCT and into survivorship.

Patients experienced social isolation, and its impact on relationships, as a consequence of the immunosuppression treatment affecting patients from the start of the treatment to a point in time afterwards [30]. The Covid pandemic was ongoing for some of the study participants, which must be taken into consideration and might have led to them keeping themselves isolated [43]. Patients’ experiences of anxiety and depressive symptoms before allo-HCT may contribute to increased negative effects of perceived social isolation [44]. Evidence-based innovative interventions before and during allo-HCT are suggested to support patients’ mental health and sense of social connectedness [44], but are also needed into survivorship [45]. Our research group has earlier described how patients prepared themselves before the transplantation in order to maintain social contacts during in-patient care, but preparedness for the post-transplant care was given less focus [46]. The impact on relationships, but also feelings of how others are affected by the patients’ treatment and recovery needs, shows the importance of involving partners and families in the care. Not least for single parents, but also for young adults who may have started to disengage and manage living independently, but then find themselves needing support, including financially.

Based on the results of this study, it is important to highlight the presence of thoughts about death experienced by patients undergoing allo-HCT. People cope with a cancer diagnosis in different ways, but thoughts about life and death often occur [24]. The current study shows that existential experiences were present for patients even after allo-HCT, something that needs to be taken into consideration in the transition to survivorship [47]. Facing a life-threatening illness and undergoing allo-HCT affect the patient’s QoL [11–15] and mean a transformative life situation in general, as does the long recovery and rehabilitation period into survivorship [11, 24, 47, 48]. Little et al. [49] describe this as ‘liminality’—a lasting state of uncertainty, disconnection, and redefined identity that begins at diagnosis. Understanding this process is key to providing meaningful care and shaping responsive healthcare systems from which patients might need support, and helping patients create a meaningful life situation [49, 50].

Identifying patient’s symptoms and challenges during and after an allo-HCT is vital, as well as in follow-up care and for cancer survivors [50–52]. Patient-reported outcome measures [53] can be useful tools to screen patients’ individual symptoms and challenges; however, the results from the present study show the importance of understanding the patient’s experience as a multidimensional whole. For this, a person-centred approach to individual patients’ experiences is needed, and for healthcare personnel to be prepared to address symptoms and challenges beyond the physical. Patients might be used to, or taught to, express physical symptoms as this is often included in various patient-reported outcome measures [53]. A follow-up conversation needs, therefore, to include a multidimensional and holistic approach to symptoms and challenges experienced by the patient to facilitate the best support. Identifying, listening to, and having conversations with patients about the side effects, symptoms or challenges that can follow allo-HCT need to start during, or even before, to help patients be prepared for the treatment and recovery [18].

### Methodological considerations

Some methodological limitations of the present study must be taken into consideration when interpreting the results. It must be noted that some of the patients were three years after allo-HCT, which could have affected their memories of their first-year experiences, representing recall bias; they may also have described experiences that were not exclusively from the first year. However, we do not believe that this would have overestimated the experiences described. We did not collect specific information about GvHD, and several of the physical symptoms or challenges could be due to this syndrome; however, we chose to not categorise presumptions of GvHD but to use the patient’s own expressions.

The study concerns some ethical aspects and, as some descriptions show, this can be a very difficult situation for patients. It needs to be clarified that the information letter stated that the study was conducted in collaboration with the allo-HCT centres, and that participation was voluntary and could be terminated at any time without explanation. Contact details of all responsible researchers were provided, and it was stated that all were registered nurses. This was intended to create an opportunity for contact if some difficulty arose. We had telephone contact with a few patients, not in connection with any symptoms or challenges but because they found the study important and were happy to take part. The online survey made it possible for the patients to reflect upon the four dimensions included and describe the symptoms and challenges they had experienced. We decided, therefore, to keep each description as written since this was the patient’s own voice. This may be regarded as an insufficiently processed result, but we view it as further illustration of how symptoms and complaints cannot be separated, showing the multidimensionality of symptoms [54]. The relatively high response rate and the fact that patients wrote long narratives might reflect a desire to share. Our sample had an equal gender distribution and a range of ages, thus strengthening transferability.

### Conclusion

This study provides insight and knowledge that shows that patients experience not only physical, but also psychological, social, and existential challenges as a multidimensional intertwined whole during the first year after allo-HCT. This knowledge emphasises the importance that healthcare professionals must be prepared to ask about, and listen to, difficult and intrusive experiences, and for patients’ needs to be assessed and addressed so that they can receive supportive care to navigate post-transplant life and survivorship.

## Data Availability

The data that support the findings of this study are available from the corresponding author on reasonable request. The data are not publicly available due to privacy and ethical restrictions.
